# A Seamless Design for the Combination of a Case–Control and a Cohort Diagnostic Accuracy Study

**DOI:** 10.1002/sim.70016

**Published:** 2025-03-05

**Authors:** Eric Bibiza‐Freiwald, Werner Vach, Antonia Zapf

**Affiliations:** ^1^ Department of Medical Statistics SGS proderm GmbH Schenefeld Germany; ^2^ Institute of Medical Biometry and Epidemiology University Medical Center Hamburg‐Eppendorf Hamburg Germany; ^3^ Basel Academy for Quality and Research in Medicine Basel Switzerland; ^4^ Department of Environmental Sciences University of Basel Basel Switzerland

**Keywords:** adaptive design, diagnostic accuracy study, research acceleration, seamless design, simulation study

## Abstract

In determining the accuracy of a new diagnostic test, often two steps are performed. In the first step, a case–control study is performed as an efficient but potentially biased design. In a second step, a population‐based cohort study is performed as an unbiased but less efficient design. In order to accelerate diagnostic research, it has recently been suggested to combine the two designs in one seamless design. In this article, we present a more in‐depth description of this idea. The seamless diagnostic accuracy study design is formally introduced by comparison with the traditional pathway, and the basic design decisions are discussed: A stopping rule and a stopping time. An appealing feature of the design is the possibility to ignore the seamless design in the final analysis, although part of the data is used already in an interim analysis. The justification for this strategy is provided by a large‐scale simulation study. The simulation study suggests also that the risk of a loss of power due to using a seamless design can be limited by a reasonable choice of the futility boundaries, defining the stopping rule. We conclude that the seamless diagnostic accuracy study design seems to be ready to use. It promises to accelerate diagnostic research, in particular if population‐based cohort studies can be started without great efforts and if the reference standard can be evaluated with little delay.

## 1 Introduction

The development of diagnostic tests is divided into different phases of clinical development, analogous to intervention studies. However, there are several phase models in the field of diagnostic studies (e.g., [1, 2, 3]), which are contrasted in Vach et al. [4] Since the development stages each focus on different objectives, the study designs of these stages also differ. In diagnostic accuracy studies (phase II in [4]), the objective is to demonstrate that the new test detects the target condition with sufficient certainty. Köbberling et al. and also the European Guideline on diagnostic agents distinguish within diagnostic accuracy studies between studies with a case–control design and confirmatory cohort studies involving a population‐based sample [1, 3]. In a case–control study, the sample size can be kept small and the case–control ratio can be set to 1:1. However, in case–control studies, the diagnostic accuracy is often overestimated, as cases and/or controls may represent more extreme situations than on average in clinical practice [5, 6]. In contrast, population‐based cohort studies provide realistic diagnostic accuracy estimates and are preferable to case–control designs [7]. At the same time, however, they require comparatively large sample sizes to estimate both sensitivity and specificity with sufficient precision, especially in the case of a very high or low prevalence [8]. Thus, case–control studies often have a gatekeeper function, where only tests with sufficient diagnostic accuracy in the case–control setting are transferred to a population‐based cohort design.

At first glance, the two study designs cannot be combined and are therefore conducted in two separate studies. Consequently, this means a high expenditure of time and money since the entire planning and all approvals are carried out twice, and sometimes years are spent between the studies, delaying clinical development.

Seamless study designs offer a way out of the general dilemma of spending much time between studies addressing different phases of clinical development. They are well‐established in therapeutic trials (e.g., [9, 10]), but such designs are also feasible and of interest in diagnostic studies [4]. Many different seamless designs are possible, and one proposed by Vach et al. allows case–control and population‐based cohort studies to be combined into one study ([4], Section 3.4). This design reduces the time until obtaining the relevant, population‐based accuracy estimates without losing the opportunity to stop early for futility.

This article aims to take a closer look at the seamless design for combining case–control and population‐based cohort studies. The additional decisions to be made—compared with performing the two studies separately—will be identified and discussed. Vach et al. pointed out that despite using part of the data in both analyses, the resulting bias is limited and allows us to perform the final analysis without any adjustments [4]. Vach et al. presented the first results of a simulation study supporting this. In this article, this investigation is extended systematically [4].

Case–control designs and population‐based cohort designs can investigate the accuracy of a single test or can compare two (or more) tests. In both cases, a seamless design is possible. In this article, we will focus on a single test design. In Section 2, the seamless design will be introduced in more detail and the simulation study will be described. Section 3 presents the results of the simulation study. Section 4 discusses the practical application of the seamless design and the missed opportunity to apply seamless designs in developing rapid antigen tests for COVID‐19. A discussion finishes the article.

## 2 Methods

### 2.1 The Traditional Statistical Methodology for Diagnostic Accuracy Studies

A diagnostic accuracy study on a single, new test compares the results of the new test with those of a reference standard that defines the true state of the target condition. Accordingly, the result of the new test, the so‐called index test, can be true positive (TP), true negative (TN), false positive (FP), or false negative (FN). It is recommended by guidelines to use sensitivity and specificity as coprimary endpoints in diagnostic accuracy studies [3]. Sensitivity is the probability of a true positive result given that a subject has the target condition, and specificity is the probability of a true negative result given that a subject does not have the target condition.

Sensitivity (se) and specificity (sp) are estimated by the number of individuals in the four subgroups defined by the test results:

$$\hat{\mathrm{se}}=\frac{n_{\mathrm{TP}}}{n_{\mathrm{TP}}+n_{\mathrm{FN}}}\;\text{and}\;\hat{\mathrm{sp}}=\frac{n_{\mathrm{TN}}}{n_{\mathrm{TN}}+n_{\mathrm{FP}}\;}.$$

In a confirmatory diagnostic accuracy trial for a single test, each endpoint should be compared individually with a predefined minimum value ($se_{0},sp_{0}$) [11]. Since sensitivity and specificity are coprimary endpoints, the global null hypothesis can only be rejected if both individual null hypotheses are rejected (intersection–union test) [12]. Accordingly, the global null and alternative hypotheses are as follows:

$$H_{0}:\mathrm{se}\leq {\mathrm{se}}_{0}\cup \mathrm{sp}\leq {\mathrm{sp}}_{0}\;\text{versus}\;H_{1}:\mathrm{se}>{\mathrm{se}}_{0}\cap \mathrm{sp}>{\mathrm{sp}}_{0}$$

To investigate the evidence for the alternative, we recommend using confidence intervals, as they are more informative than statistical tests. Their use can be linked to hypothesis testing by rejecting the null hypothesis if the lower limit of the respective two‐sided (1−α)‐confidence interval ${\mathrm{CI}}_{\mathrm{se},l}$ and $CI_{\mathrm{sp},l}$ is above $se_{0}$ or $sp_{0}$, respectively. After specifying the method for the computation of the interval (e.g., Wald, Clopper‐Pearson, or Wilson), the required sample size can be calculated separately for the number of individuals with and without the target condition (e.g., [13, 14, 15, 16]). To obtain the sample size in the final analysis, the prevalence must be considered recommend optimally splitting the power between the two hypotheses so that overpowering concerning the global hypotheses is avoided [8, 17].

### 2.2 The Basic Idea of a Seamless Design

**FIGURE 1:**
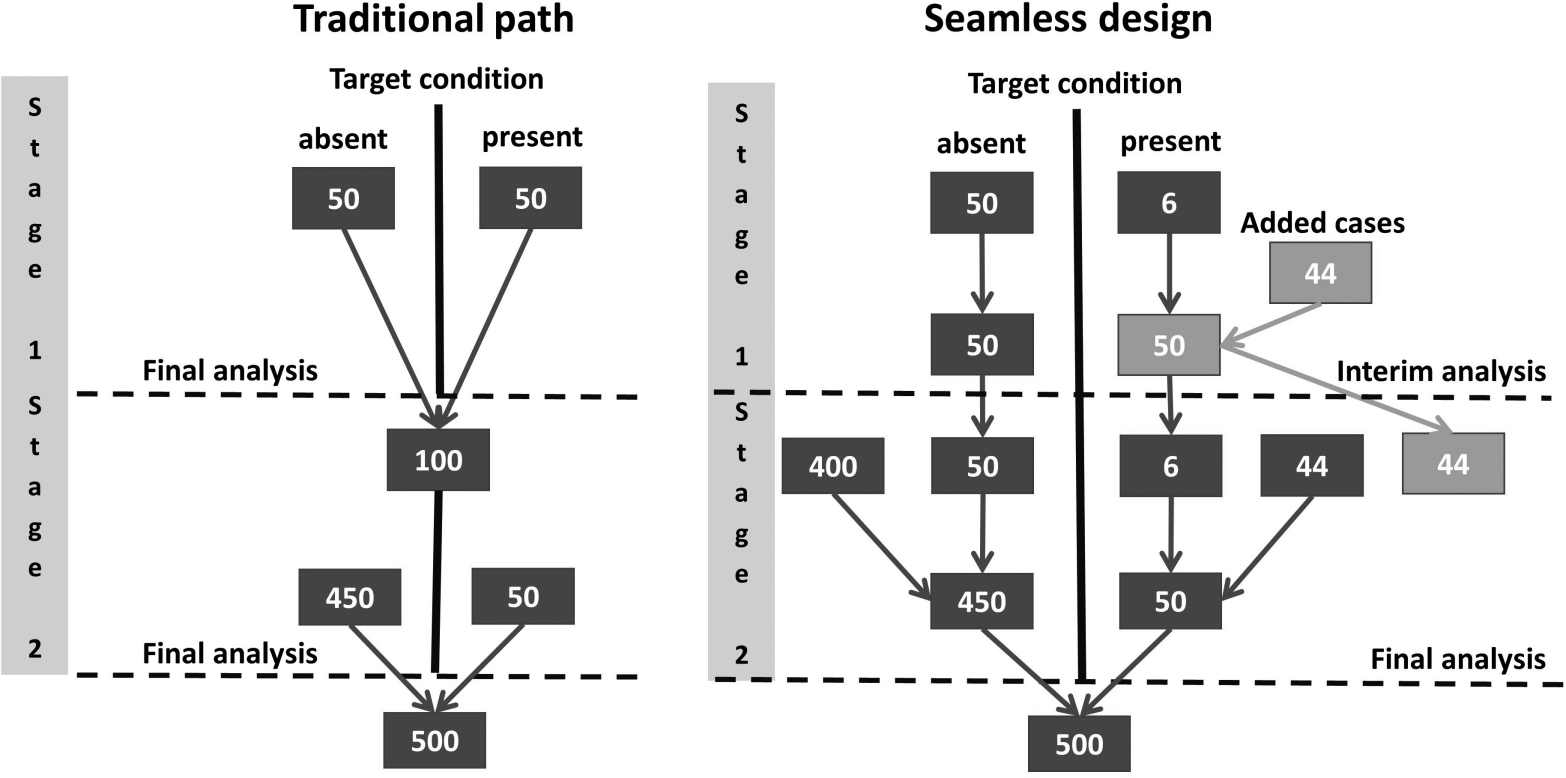
Comparison of the traditional path and the seamless design for an example scenario with a prevalence of 10% and a sample size of 100 individuals for the case–control study/interim analysis at the end of stage I and 500 individuals for the cohort study/the final analysis at the end of stage II.

Following the traditional path of diagnostic research, the first step is to perform a case–control study. The data would be analyzed, and if the diagnostic accuracy is sufficiently high, a confirmatory cohort study with a population‐based sample would then be conducted. In contrast, the seamless design would recruit population‐based from the beginning. Once a certain number of participants have been recruited, as many individuals with or without the target condition are added (e.g., from retrospective data sources), so that the groups of cases and controls are of equal size. Based on these data, a decision is made whether to continue the study or to stop early for futility. If continued, the added individuals are removed in the final analysis, such that a valid population sample informs the final estimates. This design will be referred to as a “seamless diagnostic accuracy design” in the following. The traditional path and the seamless design are contrasted in Figure 1 for an example scenario with a target condition prevalence of 10%. In the sequel, we consider the case of a prevalence below 50%, such that we always have to add cases and not controls. The case of a prevalence above 50% can be handled analogously by adding controls.

In addition to the time benefits, the savings for study participants also become apparent (here 600 in the traditional workflow versus 500 plus 44 added in the seamless design).

In the interim analysis, only stopping for futility is of interest (if the estimates indicate a diagnostic accuracy beyond the minimal values of interest). It would make little sense to stop for success, as adding cases from other sources makes it likely to obtain overoptimistic estimates of the diagnostic accuracy. Consequently, from the testing perspective, there is no need to adjust significance levels in the final analysis to control the type 1 error rate [8]. However, the double use of part of the data in the interim analysis and the final analysis may imply a (conditional) bias in the final accuracy estimates, as the final analysis is only reached if the interim analysis has indicated sufficient accuracy. In a simulation study, we will demonstrate later that this bias is negligible and does not invalidate the statistical inference of the final analysis. Hence, the final analyses can be performed the same way as in the traditional path. Consequently, the sample size for the final analysis can be determined in the usual way and does not require any specific considerations.

To describe the seamless design completely, only two further specifications are necessary: A stopping rule and the time point of performing the interim analysis.

### 2.3 Choice of Stopping Rule

**FIGURE 2:**
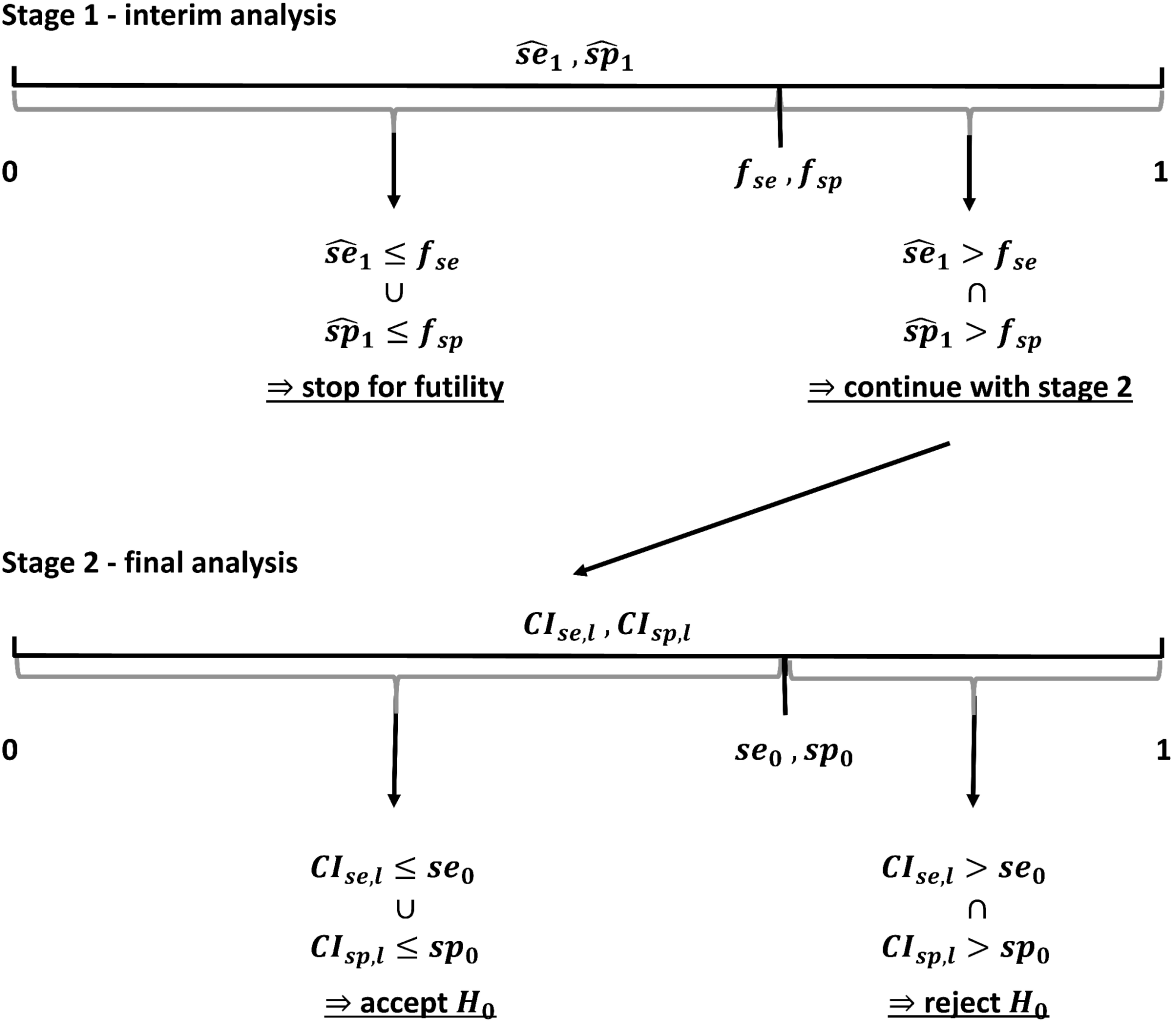
Decision criteria in the two study parts by comparison of the estimators and the futility boundaries in the interim analysis of stage I (${\hat{\mathrm{se}}}_{1}$ vs. $f_{\mathrm{se}}$ and ${\hat{\mathrm{sp}}}_{1}$ vs. $f_{\mathrm{sp}}$) and of the lower limits of the confidence intervals and minimum sensitivity and specificity in the final analysis of stage II ($CI_{\mathrm{se},l}$ vs. $se_{0}$ and $CI_{\mathrm{sp},l}$ vs. $sp_{0}$).

A stopping rule can be specified by defining futility boundaries $f_{\mathrm{se}}$ and $f_{\mathrm{sp}}.$ At the interim analysis at the end of stage I, the study should be stopped for futility if one of the point estimators ${\hat{\mathrm{se}}}_{1}$ and ${\hat{\mathrm{sp}}}_{1}$ or both are below the corresponding futility bound. Only if both estimators are above the corresponding futility boundaries, we proceed with stage II. In the final analysis at the end of stage II, the confirmatory decision about accepting or rejecting the null hypotheses is based on the comparison of the lower limits of the confidence intervals for sensitivity and specificity $CI_{\mathrm{se},l}$ and $CI_{\mathrm{sp},l}$ and the predefined minimum sensitivity and specificity $se_{0}\;\text{and}\;sp_{0}$. The resulting decision criteria in the parts of the seamless design are shown in Figure 2.

The choice of the futility boundaries depends on several factors. In any case, they should be lower than the minimally acceptable values for sensitivity and specificity, that is, at which the new test may still be regarded as valuable. The lower the futility boundaries, the greater the risk of continuing even if a test has an accuracy below the minimally acceptable values. The higher the futility boundaries, the greater the risk of stopping at the interim analysis for futility, even if a test has an accuracy (slightly) above the minimally acceptable values. The choice of the futility boundaries may also depend on the population size to be included in the interim analysis (i.e., the stopping time) or the type of cases added. Due to these multiple, conflicting issues, it is hard to specify a formal procedure to determine the futility boundaries. We expect that futility boundaries will often be chosen slightly below the minimally acceptable values but distinctly below the expected sensitivity and specificity. This way, it can be ensured that a test corresponding to its expectations will reach the final analysis but that there is a chance for early stopping in case of a distinct failure.

### 2.4 Choice of Stopping Time

A seamless design with two stages requires to decide when to stop the first stage and to perform the interim analysis. We assume that there is an intended sample size for the population‐based study to be analyzed finally and that hence the stopping time finishing stage I can be expressed as a fraction s of this intended sample size. The stopping time should be sufficiently large to ensure a sufficient precision of the estimates in the interim analysis, but it should be not too large to avoid the waste of resources in case of futility. The absolute number of available cases or time constraints for stage I can also play a role. We expect that such considerations will typically result in stopping times between 0.1 and 0.3. In Appendix A, we illustrate that sample size considerations for the analysis to be performed in the interim analysis will also often support such a choice.

### 2.5 Composition of Analysis Population in the Interim Analysis

Table 1 illustrates how the choice of the stopping time together with the intended sample size for the final analysis and the prevalence determines the composition of the population analyzed in the interim analysis. It should be noted that due to the low prevalence, the added cases will dominate the population of cases. This implies that the analysis performed in the interim analysis will be close to the analysis of a case–control study with controls already representative of the patient population.

**TABLE 1:** The composition of the analysis population in the interim analysis in dependence on the prevalance, the sample size in the final analysis, and the stopping time *s*. The sample sizes chosen for the final analysis reflect the situations to have 100 or 50 cases in the final analysis, respectively.

Prevalence	Sample size in final analysis	Stopping time s	Cases from the population‐based sampling	Added cases	Controls
0.1	1000	0.1	10	80	90
		0.2	20	160	180
		0.3	30	240	270
	500	0.1	5	40	45
		0.2	10	80	90
		0.3	15	120	135
0.2	500	0.1	10	30	40
		0.2	20	60	80
		0.3	30	90	120
	250	0.1	5	15	20
		0.2	10	30	40
		0.3	15	45	60
0.3	333	0.1	10	13	23
		0.2	20	27	47
		0.3	30	40	70
	167	0.1	5	7	12
		0.2	10	13	23
		0.3	15	20	35

### 2.6 Application Example

**FIGURE 3:**
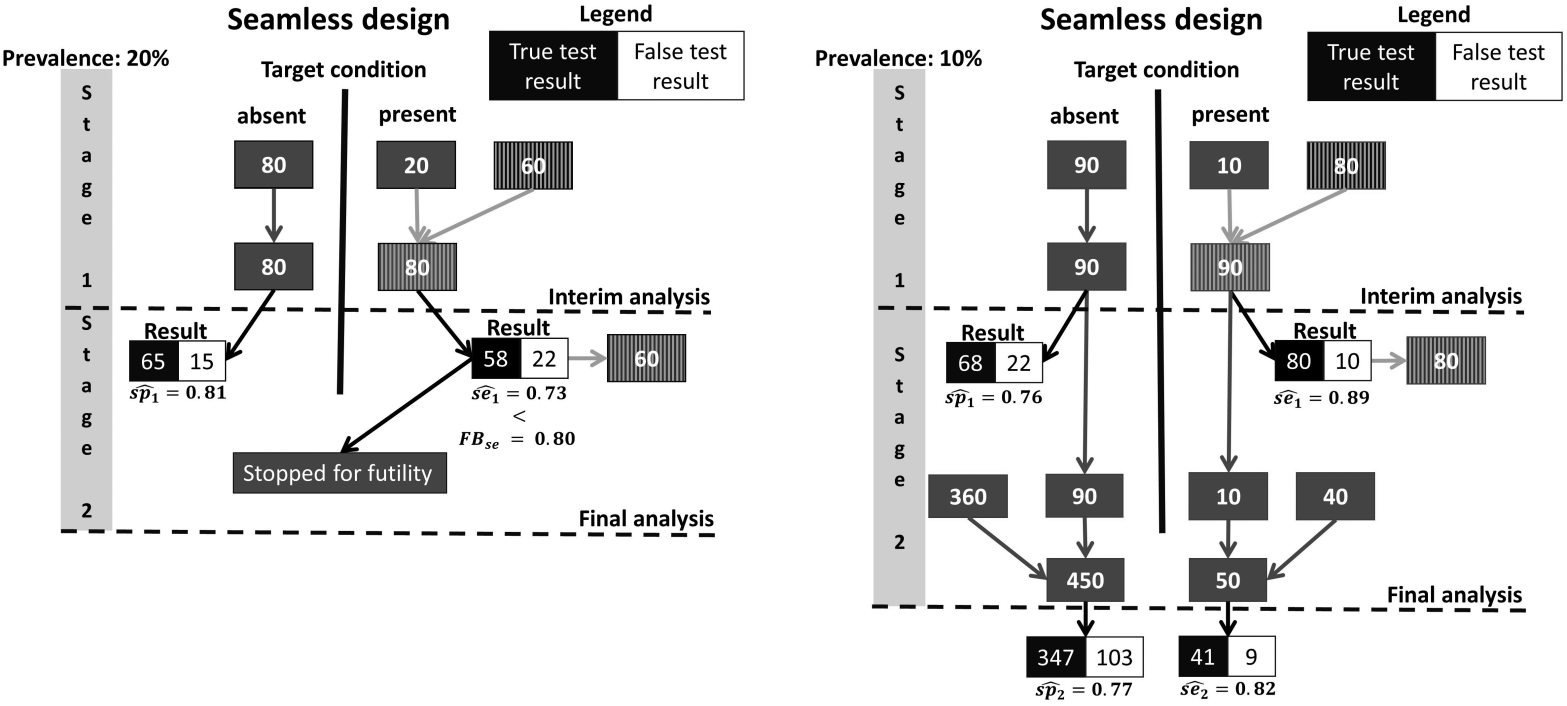
Illustration of two hypothetical applications of a seamless design. The left‐hand course of the study demonstrates a stop due to futility because the sensitivity was estimated too low after the end of the first stage. The right‐hand course of the study demonstrates a scenario in which sufficiently good results have been recognized in each stage and which has been successfully completed accordingly.

Figure 3 illustrates the application of the seamless design by two hypothetical studies. In both studies, it was desirable to reach a sensitivity of $se_{0}=90\%$ and a specificity of $sp_{0}=85\%$ for the index test. The futility boundaries were chosen 10 percentage points below the desired values, that is, $FB_{\mathrm{sp}}=0.80$ and $FB_{\mathrm{se}}=0.75$.

In the population‐based cohort study, we expected a prevalence of π=20%, suggesting an overall sample size of N=500 in order to obtain n=100 cases, allowing us to estimate the desired sensitivity of $\mathrm{se}n_{0}=0.9$ with a standard error of $SE_{\mathrm{sen}}=0.03$. The stopping time was chosen as s=0.2; that is, an interim analysis was planned after including n=100 patients in a population‐based cohort. With the expected prevalence of π=20%, we expect to need $n_{\mathrm{add}}=60$ additional cases to reach an equal number of cases and controls at the interim analysis.

In the first study (Figure 3, left side), the expected prevalence was met rather precisely in stage 1. In the interim analysis, the sensitivity of $\hat{\mathrm{se}n_{1}}=0.73$ and the specificity of $\hat{\mathrm{sp}e_{1}}=0.81$ were observed, that is, a sensitivity less than the specified futility boundary. Consequently, the study was stopped for futility. In the second study (Figure 3, right side), the prevalence in stage 1 was only $\hat{\pi }=10\%$, so $n_{\mathrm{add}}=80$ additional cases were needed. Here, in the interim analysis, the sensitivity of $\hat{\mathrm{se}n_{1}}=0.89$ and the specificity of $\hat{\mathrm{sp}e_{1}}=0.76$ were observed. As both estimates were above the corresponding futility boundaries, the study was continued. The final estimates of sensitivity and specificity were $\hat{\mathrm{se}n_{2}}=0.82$ and $\hat{\mathrm{sp}e_{2}}=0.77$, suggesting that the additional cases of stage 1 were not representative of the population of interest.

### 2.7 Simulation Study

To investigate the properties of the seamless design systematically, a wide range of scenarios defined by the combination of seven different choices was considered (Table 2). We did not assume any specific rules for choosing the sample size for the final analysis, the stopping time s, or the futility boundaries, as typically the prior knowledge about sensitivity, specificity, and prevalence is fragile in diagnostic accuracy studies, and the final results (including prevalence) often contradict the prior assumptions. Hence, considering a wide range of scenarios allowing different possible combinations seems to be more informative.

**TABLE 2:** The seven choices considered in defining the simulation designs.

Sample size in the final analysis N	333, 500, 1000
Stopping time s expressed as the proportion of the sample size for the final analysis of stage II	0.1, 0.2, 0.3
Prevalence p	0.1, 0.2, 0.3.
True sensitivity	0.65, 0.75, 0.85, 0.95
True specificity	0.65, 0.75, 0.85, 0.95
Distance Δ between the futility boundary and true sensitivity and specificity (futility boundaries minus true values). The same distance was assumed for sensitivity and specificity	−0.3, −0.25, −0.2, −0.15, −0.1, −0.05, 0.0, 0.05, 0.1
Increase in sensitivity for the added cases	0.0, 0.1

With the combination of all possible choices, 7776 scenarios have been investigated in the simulation study. For each scenario, 40000 simulation runs are performed. As recommended by Morris et al. the evaluation is based on important statistical properties, such as bias, coverage probability, and statistical power [18]. How the parameters were calculated is shown in Table 3. Note that in computing the conditional relative bias, the bias is related to 1 minus the true values. This reflects that the same bias in absolute terms gets more harmful the closer the true values are to 1.0.

**TABLE 3:** Calculation of the performance measures in the simulation study.

Performance measure	Calculation method per scenario
Probability of early stopping	Relative frequency of stopping after case–control part, that is, the proportion of early stopped simulation runs out of all simulation runs.
Conditional relative bias	Mean difference of the estimated sensitivity and specificity (in the final analysis) and the corresponding true values, divided by 1 minus the true values and multiplied by 100. This calculation is based only on those simulation runs without stopping early for futility.
Conditional coverage probability	Number of simulation runs where the confidence interval covered the true sensitivity or specificity, respectively, in the final analysis, divided by the total number of simulation runs without stopping early for futility.
Unconditional coverage probability	Number of simulation runs where the confidence interval covered the true sensitivity or specificity, respectively, in the final analysis, divided by the total number of simulation runs. In case of early stopping for futility, the corresponding run is counted as covering the true value.
Statistical power	Number of simulation runs where the global null hypothesis was rejected in the final analysis divided by the total number of simulation runs. In case of early stopping for futility, the corresponding run is counted as not rejecting the null hypothesis. (For this part, 25 different null hypotheses were considered reflecting all combinations of the values 0.55, 0.65, 0.75, 0.85, and 0.95 for hypothetical true values $se_{0}$ and $sp_{0}$ to be tested.)
Type I error	Identical to statistical power, but restricted to the case of equality between the true values and the hypothetical true values to be tested.

As pointed out above, the main concern is a potential bias in the estimates of sensitivity and specificity when reusing part of the data from stage I in the final analysis of a seamless design. However, the bias may be negligible if the overlap between the populations evaluated in the interim and the final analysis is small. The stopping time s is hence expected to play a role: the lower the value of s, the less bias can be expected. Furthermore, due to removing the additional cases, there is less overlap in the cases than in the controls. Hence, specificity is expected to be more affected than sensitivity. Another important factor is the futility boundary. The lower the futility boundary, the less likely it is to stop for futility, and the less bias has to be expected.

However, potential bias is only one concern. Early stopping may also invalidate the estimation of standard errors. However, as early stopping implies a truncation of the distribution of the case–control estimates, the naïve standard errors of the final analyses are expected to overestimate the true ones, resulting in a conservative behavior of confidence intervals.

A further general concern about allowing early stopping is the adequateness of the stopping frequency. Stopping rates should be high if true sensitivity and specificity are below the futility bounds and low if they are (distinctly) above. In addition, early stopping for futility should not happen in scenarios with high power to reject the global null hypothesis of interest when following the traditional path.

The simulation study was conducted using Stata 15.1. For the computation of confidence intervals, the Wilson‐score approach was used, as recommended by Newcombe [15, 19].

## 3 Results

### 3.1 Probability of Early Stopping

**FIGURE 4:**
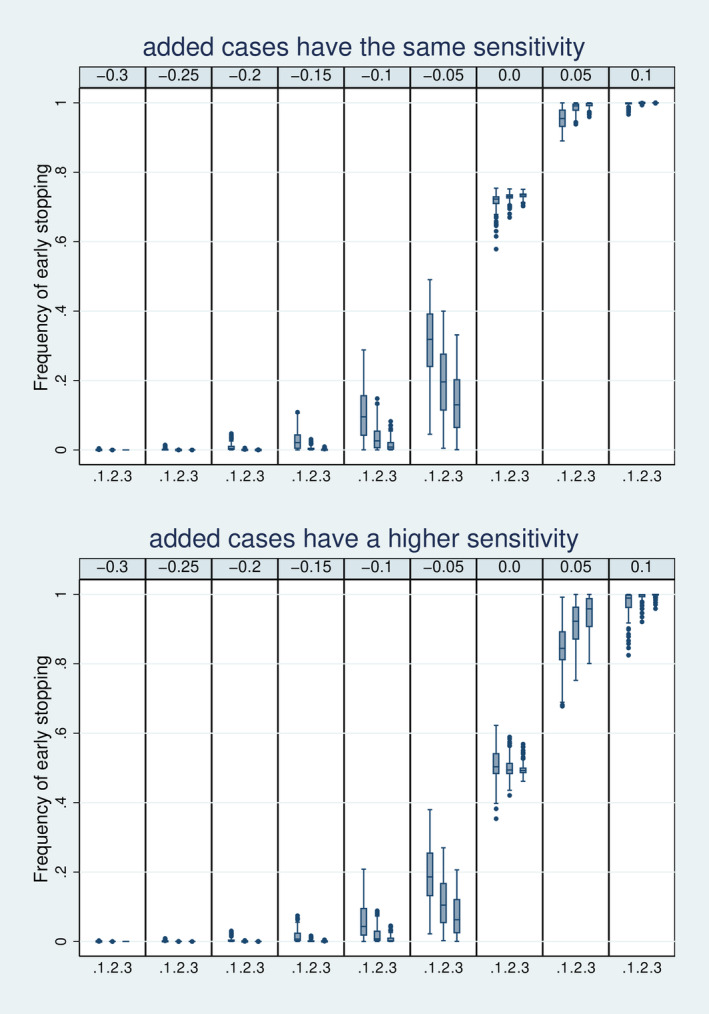
Distribution of the frequency of early stopping over all 7776 simulation designs stratified by s=(0.1,0.2,0.3) and Δ = (−0.3,…, 0.1).

Figure 4 depicts the distribution of the frequency of early stopping in dependence on Δ and stopping time s. The figure indicates that these two values determine the frequency of early stopping to a high degree, which in addition, depends on the scenario chosen for the sensitivity of the added cases. In the upper part of the figure, the case of no increase in sensitivity is considered. If the true values of sensitivity and specificity coincide with the futility boundaries (Δ=0), the frequency of early stopping is around 75%, reflecting that the probability of each estimate to be above the true value is close to 50%. If the true values are 0.05 below the futility boundaries, the frequency of early stopping is usually above 90%, indicating a high efficiency in sorting out this unfavorable situation. As soon as the true values are 0.1 below the futility boundaries, early stopping becomes almost sure, in particular, if s is 0.2 or above. In general higher values of s imply a higher frequency of early stopping if the true values are below the futility boundaries and a lower frequency of early stopping if the true values are above the futility boundaries. This underlines that higher values of s increase the efficiency of the seamless design. The lower part of Figure 4 depicts the case of increased sensitivity for the enriched cases. As expected, the frequency of early stopping decreases, and we can be less confident that we stop early in the case of true futility (i.e., Δ>0).

### 3.2 Conditional Relative Bias

**FIGURE 5:**
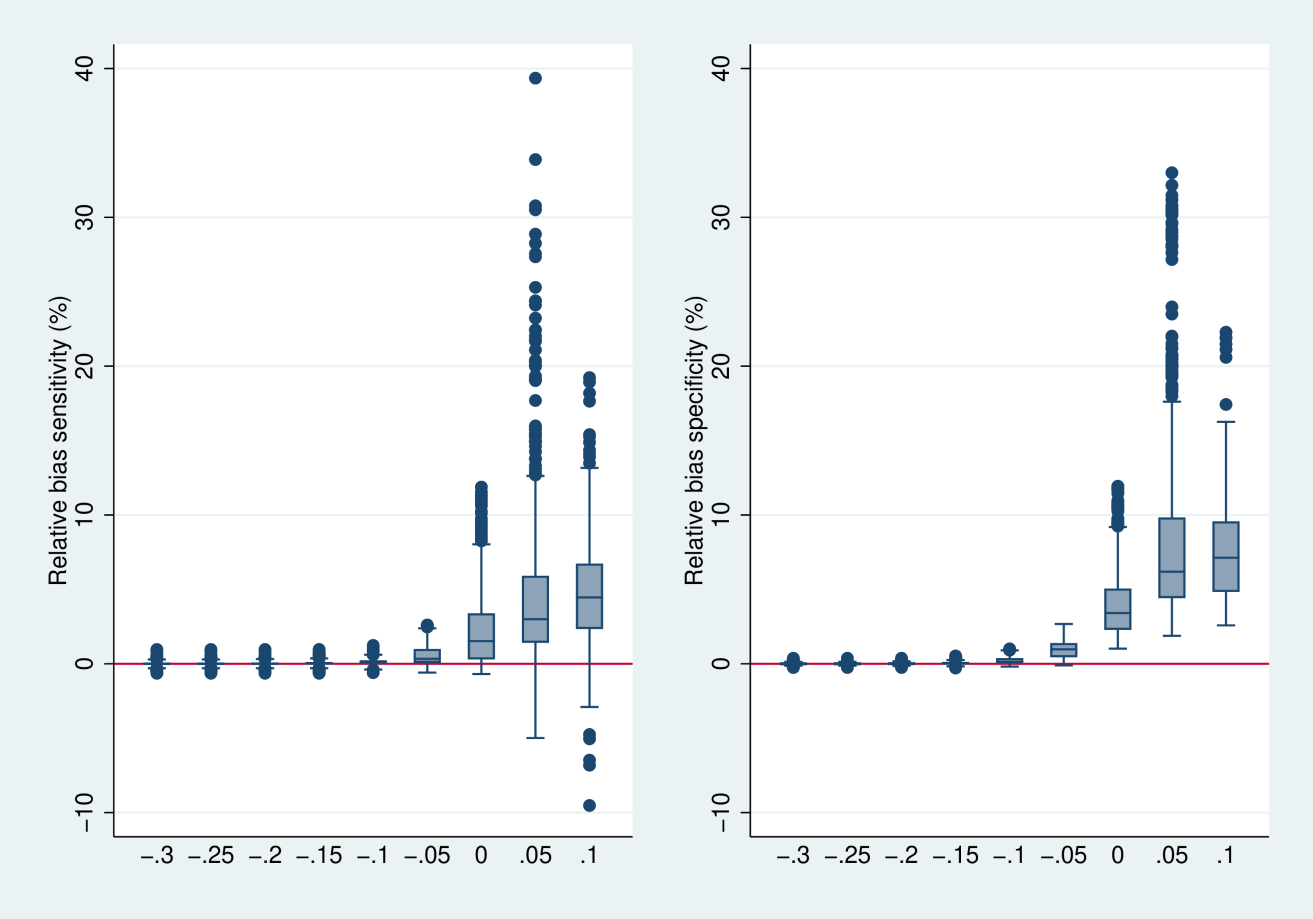
Distribution of the relative bias over all 7776 simulation designs stratified by. However, 693 designs with a stopping frequency above 0.999, that is, with less than 40 final analyses performed, are omitted.

Figure 5 depicts the distribution of the conditional relative bias in dependence on Δ. As expected, the bias tends to be positive and more pronounced on average for specificity than for sensitivity. If Δ is low, there is little bias as the frequency of stopping is very low (cf. Figure 4), and with increasing values of Δ the bias increases, as the frequency of stopping increases. It can be observed that the relative bias is typically limited to 2.5% if the true values exceed the futility (Δ<0) and to 10% in the case of Δ=0. If the case of Δ>0, the relative bias can become more substantial, probably reflecting the high frequency of early stopping.

**FIGURE 6:**
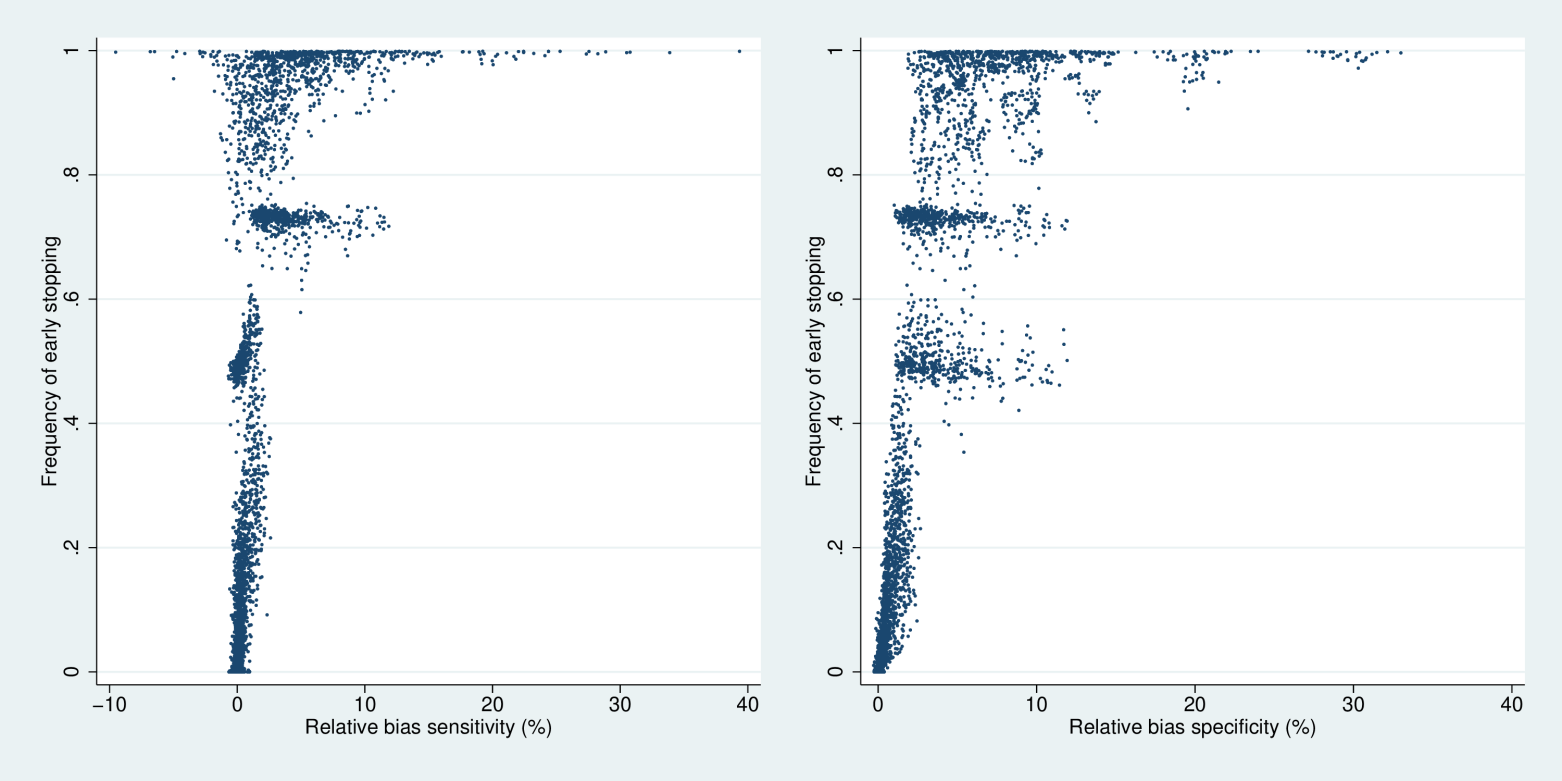
Joint distribution of relative bias and stopping frequency over all 7776 simulation designs. However, 693 designs with a stopping frequency above 0.999, that is, with less than 40 final analyses performed, are omitted.

Figure 6 directly depicts the relation between relative bias and the frequency of stopping. It illustrates that a considerably high relative bias in specificity appears only in designs with a very high frequency of stopping, typically above 0.9. In designs with a stopping frequency below 80%, the relative bias is mostly limited to 10%. The results are even more favorable with respect to the relative bias in sensitivity.

As expected, the relative bias increases with increasing stopping time. For example, in the case of Δ=0, the 90th percentile was 5.3 for s=0.1, 7.6 for s=0.2, and 9.3 for s=0.3.

### 3.3 Coverage Probability

**FIGURE 7:**
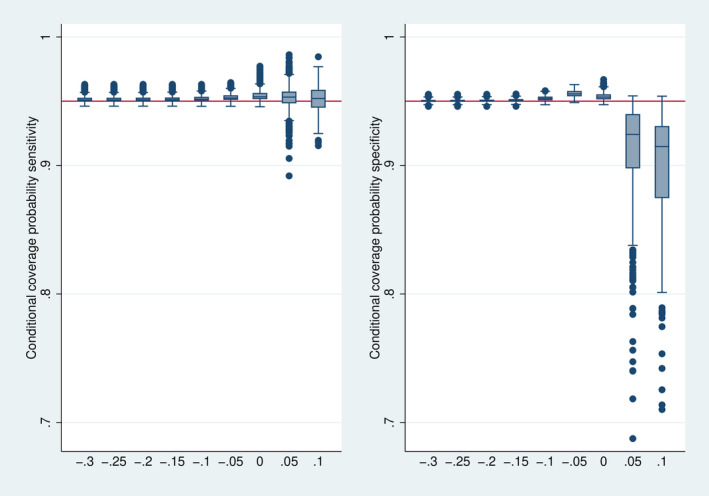
Distribution of the conditional coverage probability over all 7776 simulation designs stratified by Δ. However, 874 designs with a stopping frequency above 0.995, that is, less than 200 final analyses performed, are omitted.

**FIGURE 8:**
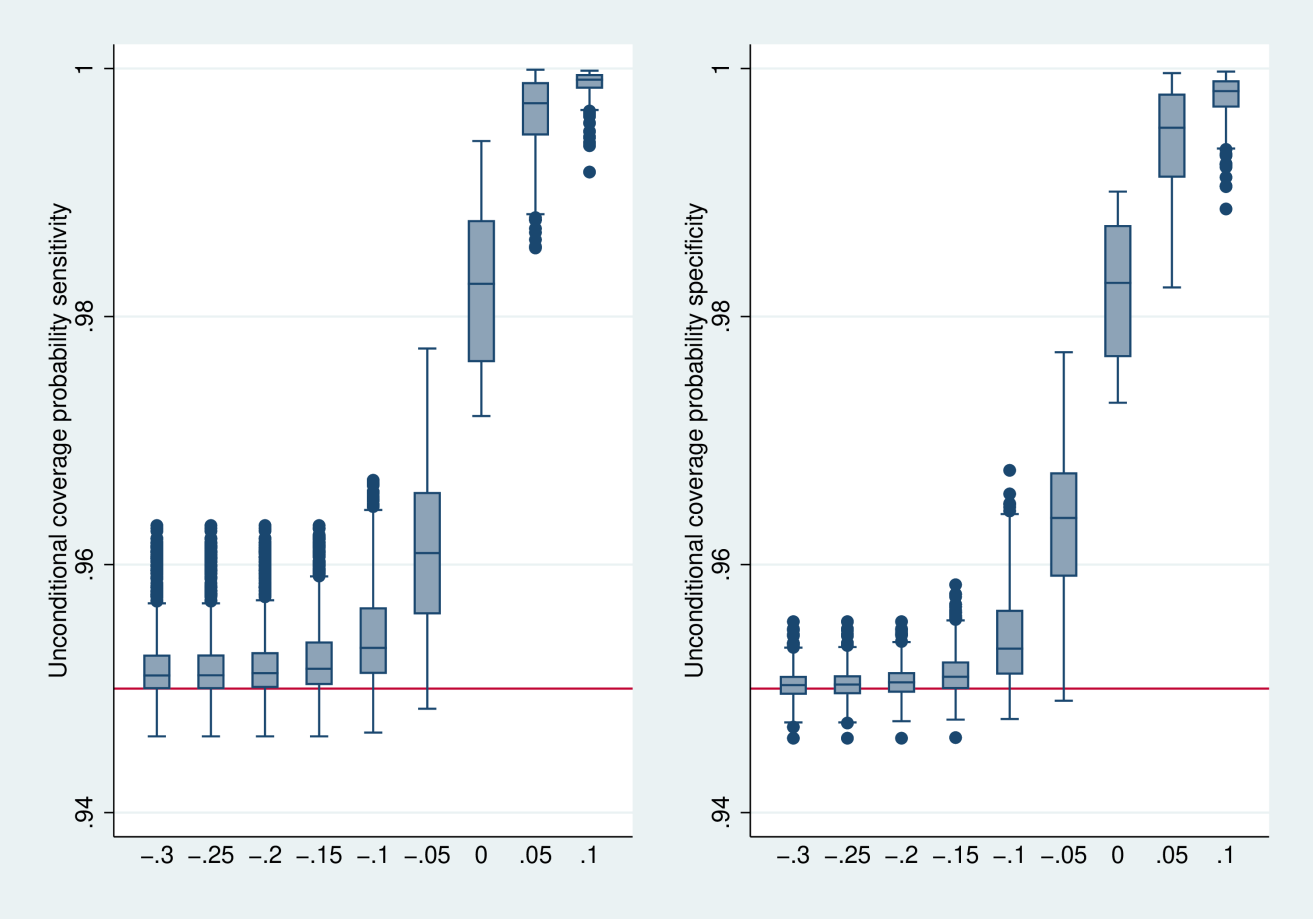
Distribution of the unconditional coverage probability over all 7776 simulation designs stratified by Δ.

Figure 7 depicts the distribution of the conditional coverage probability. The confidence intervals show mainly a conservative behavior, that is, the true coverage is above the nominal level of 95%. This corresponds to the expectation expressed above. Only if Δ is above 0 it may happen that the bias starts to play an essential role resulting in an anticonservative behavior, in particular for specificity. When considering the unconditional coverage probabilities (Figure 8), the early stopping implies a conservative behavior over the whole range.

### 3.4 Loss of Power

To address the question of a loss of power when starting directly with a seamless design, the power of the seamless design is compared with the power of a population‐based study with the same sample size in the final analysis. In this comparison, we ignore that a case–control study in the traditional path may have already implied a stop for futility. We did this as there is wide liberty in setting up such a case–control study.

**FIGURE 9:**
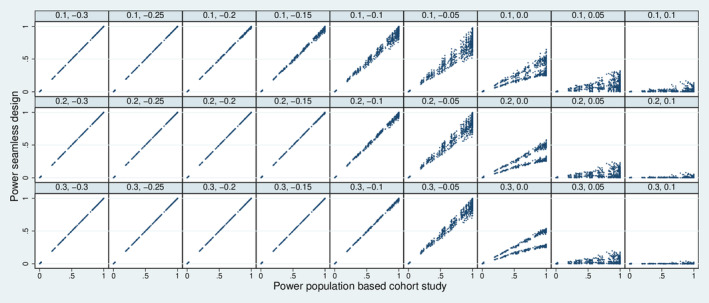
The joint distribution of the power of the seamless design and the power of a population‐based cohort study over all 7776 simulation designs combined with all 25 choices for $se_{0}$ and $sp_{0}$ stratified by s (rows) and Δ (columns).

Figure 9 depicts the joint distribution of the power of the seamless design and the power of the population‐based cohort study. As expected, a seamless design can decrease the power. This decrease can be substantial if the true values are close to or below the futility boundaries. However, a value of Δ of −0.1 or below implies nearly a protection against a loss of power, in particular, if the stopping time s is 0.2 or above.

### 3.5 Type 1 Error Rate

**FIGURE 10:**
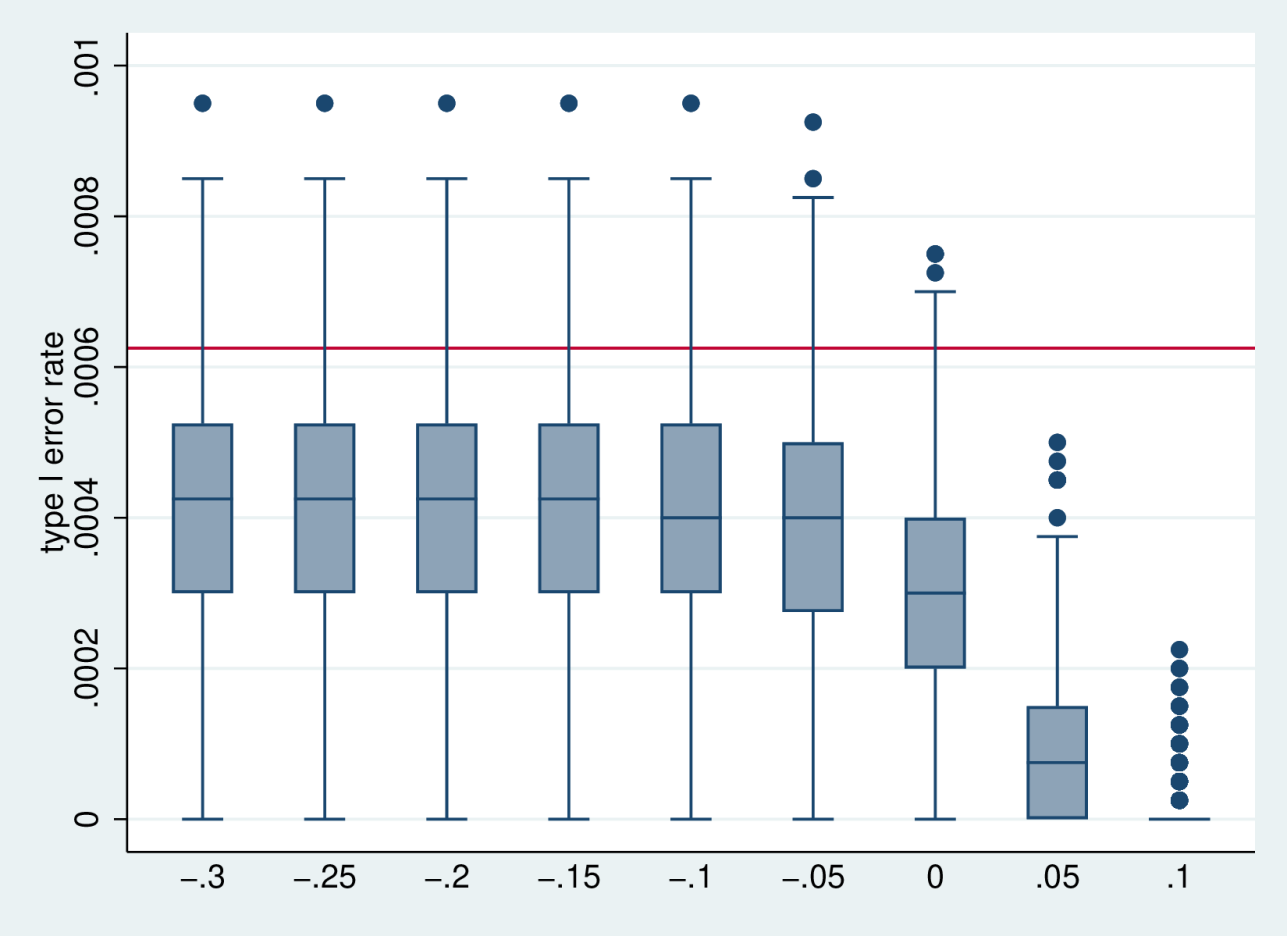
Distribution of the type I error rate over all 7776 simulation designs stratified by Δ.

Figure 10 depicts the distribution of the type I error rate. Since we combine two stochastically independent tests at the 2.5% level by the union intersection principle, the nominal level of the test is 0.000625. The actual level seems to be slightly lower on average, and it decreases further in the case Δ≥0, that is, if the frequency of stopping increases, as early stopping implies a failure to reject the null hypothesis.

## 4 The Practical Application of the Seamless Design

A seamless design has already been used in the study of Jensen et al. [20] That study aimed at evaluating a new questionnaire‐based screening test for lumbar spinal stenosis in a cohort of patients approaching a secondary care spine clinic. The expected prevalence was 15%, suggesting a sample size of several 100 patients. The research group hence decided to collect simultaneously also additional cases from two surgical departments, namely patients scheduled for decompression surgery due to LSS. The group aimed to include 100 cases and 100 controls in an interim analysis, but due to practical circumstances, the interim analysis was performed after the inclusion of 107 cases and 138 controls. The observed specificity for a cutpoint implying a sensitivity of 95% was below the prespecified futility boundary for the specificity of 68%, and the study was stopped for futility.

There are two practical aspects that were very favorable for applying a seamless design in this example. First, a population‐based cohort was directly accessible to the researchers developing the test. However, this may not be the case for all test developers. In particular, for laboratory tests, it might be much easier for the test developer to use existing samples from the developer's biobank of the lab than to establish a collaboration with clinicians providing access to a cohort for which the application of the test is of clinical interest. Second, the reference status of a patient could be determined immediately by the clinicians at a patient's visit. This needs not to be the case for all diagnostic problems.

An interesting hypothetical example is the development of rapid antigen tests for COVID‐19. The World Health Organization (WHO) defined 80% sensitivity and 97% specificity as minimum criteria [21]. In Germany, most manufacturers have received the CE mark for their tests under the accelerated process. Even though in the case–control studies most of the tests met this criterion (79% of all investigated tests, according to PEI), this was mainly because patients with severe symptoms were used as cases and pre‐Corona blood donors as controls [22, 23]. In subsequent cohort studies, the sensitivity was considerably lower in symptomatic individuals (73% sensitivity on average), and even lower in asymptomatic individuals with 54.7% on average [23].

In this context, it would have been realistic to start population‐based studies in symptomatic individuals or even in asymptomatic individuals rather early, as the corresponding cohorts were existing and the results from the reference standard (PCR) were available with a short delay. Using this opportunity and combining it with a seamless design might have allowed having realistic estimates rather rapidly without losing the possibility of identifying futile tests very early.

## 5 Discussion

### 5.1 The Statistical Feasibility of the Seamless Diagnostic Accuracy Study Design

In this article, we made the first concrete suggestion for a seamless diagnostic accuracy study design when the aim is to determine the diagnostic accuracy of a single test. The application of this design seems to be feasible. It has the attractive feature that there is no need to consider the specific design in the final analysis. This was demonstrated in a large‐scale simulation study, indicating a non‐negligible bias and invalidity of confidence intervals only under rare circumstances, that is, when by chance a study is not stopped for futility, although the probability for stopping is very high.

The use of the seamless design requires specifying futility boundaries for sensitivity and specificity and a stopping time on the time scale of recruited patients. For both decisions, conflicting requirements can be identified, which makes the use of explicit rules to determine these numbers cumbersome. Our investigations suggest that it is reasonable to choose the futility boundaries at least 10 percentage points below the expected or desired values, and to prefer stopping times between 20% and 30%. With such a choice, the risk of overlooking a promising new test due to a false early stopping is low while the risk of bias is still limited.

### 5.2 The Practical Feasibility of the Seamless Diagnostic Accuracy Study Design

As pointed out in the introduction, seamless designs can contribute to accelerating research, as population population‐based studies and case–control studies can start together. However, this implicitly assumes that population‐based studies can be started simultaneously when case–control studies can start. In principle, this is always possible, as relevant clinical populations do exist. However, they may not be easily accessible to the test developer. Seamless designs are most attractive if diagnostic tests are developed in a collaborative effort involving all relevant stakeholders, and the additional efforts to establish a population‐based cohort are limited. However, even if establishing a population‐based cohort may imply some delay, this delay will often still be slight compared to starting a population‐based study after finishing a case–control study.

In our considerations about the seamless design, we made further the implicit assumption that the reference status of a patient can be determined immediately or with only a short delay. This need not be the case for all diagnostic problems. If determining the reference status requires following the patient's clinical course for some time, seamless designs may become less attractive, as the population‐based sampling has to be continued until the data of the patients included in the interim analysis can be analyzed.

### 5.3 Further Steps

In this article, we considered the specific case of a diagnostic accuracy study on a single test. However, a seamless design can also be used for comparative studies comparing two diagnostic tests, using differences in sensitivity and specificity as the relevant parameters. It can also be used to evaluate or compare continuous diagnostic markers using for example the area under the ROC curve as the relevant parameter for decision making. All these extensions require corresponding guidance for choosing the values of the futility boundaries and the stopping time.

Specifying futility boundaries separately for sensitivity and specificity seems to be adequate if the analysis of the diagnostic accuracy study is based on a separate evaluation of sensitivity and specificity. However, an analysis can also be based on considering prespecified weighted combinations [24]. In this case, it might be more adequate to consider one futility boundary for a prespecified combination, for example, the Youden index, closest‐to‐(0,1) criterion or Liu's method [25, 26, 27].

The considerations in this article present a first look at the simplest version of a seamless design for a diagnostic accuracy study. Further refinements of this design are possible. The first example is an attempt to investigate the potential bias introduced by selecting the added cases from another source. If the sensitivity estimated from the added cases is distinctly larger than the sensitivity estimated from the population‐based cases, more weight may be given to the latter estimate. However, such considerations require a sufficient number of population‐based cases, which may require to increase in the stopping time (see Table 1). A second example is a reassessment of the sample size after the interim analysis, considering the current estimates of the prevalence and diagnostic accuracy [8]. The value of such a reassessment is not completely obvious, as a low prevalence cannot be estimated precisely from the interim analysis, and accuracy estimates are partially biased. A reassessment may also impact the properties investigated in the simulation study presented in this article, as the value of the stopping time *s* changes in dependence on the interim analysis results.

### 5.4 Limitations of This Study

Although we performed a large‐scale simulation study, the study could not cover all potential scenarios. We assumed that it is possible to add sufficient cases for the interim analysis to obtain a complete balance. This need not be the case in practice, as illustrated by the cited study of Jensen et al. [20]. We also considered only the specific situation that the futility boundaries for sensitivity and specificity are at the same distance to the true values. In Appendix B, we present additional results for the case of unequal distances, suggesting that this is not a major limitation.

### 5.5 Outlook and Conclusions

In our opinion, the suggested seamless diagnostic accuracy design is ready to use for the specific case of investigating the accuracy of a new, single test.

In the case of a new test with promising diagnostic accuracy, we can see two major advantages:

- The delay between two study phases, which can sometimes be years, is reduced to an absolute minimum. This can accelerate the research process enormously.
- There is only a need to set‐up one study, reducing the amount of necessary resources. The need to include a lower number of patients overall may imply a further reduction.

In the case of a new futile test, we may have to pay the price that setting up a population‐based study requires more effort and resources than setting up a case–control study. However, we still have the advantage of being closer to a population‐based study and may stop for futility, even if a case–control study would have suggested continuing.

Considering the advantages of a test with promising diagnostic accuracy, we consider it worthwhile to consider the use of a seamless design for any new test. Increasing practical experience with this design and additional methodological refinements and extensions can contribute to the desirable widespread use.

## Conflicts of Interest

The authors declare no conflicts of interest.

## Figures and Tables

**FIGURE B1:**
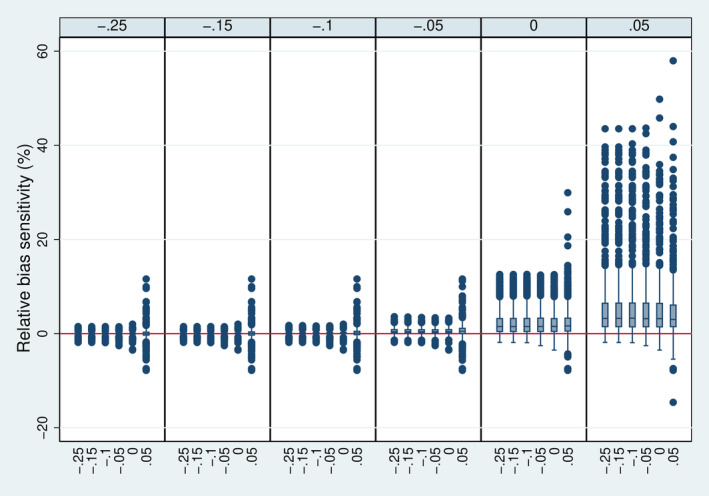
Distribution of the relative bias for sensitivity over all 31 104 simulation designs stratified by $\Delta_{\mathrm{se}}$ (top numbers) and $\Delta_{\mathrm{sp}}$ (bottom numbers). However, 749 designs with a stopping frequency above 0.995, that is, with less than 50 final analyses performed, are omitted.

**FIGURE B2:**
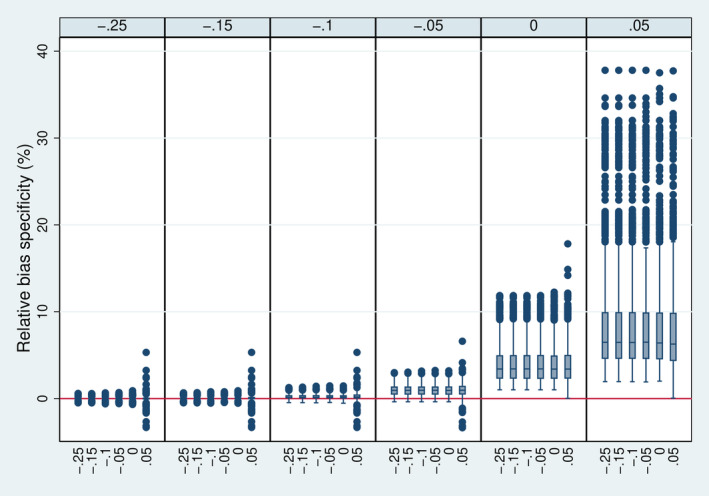
Distribution of the relative bias for specificity over all 31 104 simulation designs stratified by $\Delta_{\mathrm{sp}}$ (top numbers) and $\Delta_{\mathrm{se}}$ (bottom numbers). However, 749 designs with a stopping frequency above 0.995, that is, with less than 50 final analyses performed, are omitted.

**FIGURE B3:**
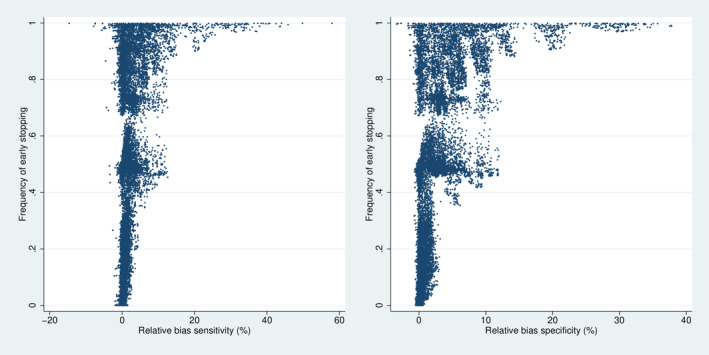
Joint distribution of relative bias and stopping frequency over all 31 104 simulation designs. However, 749 designs with a stopping frequency above 0.995, that is, with less than 50 final analyses performed, are omitted.

**FIGURE B4:**
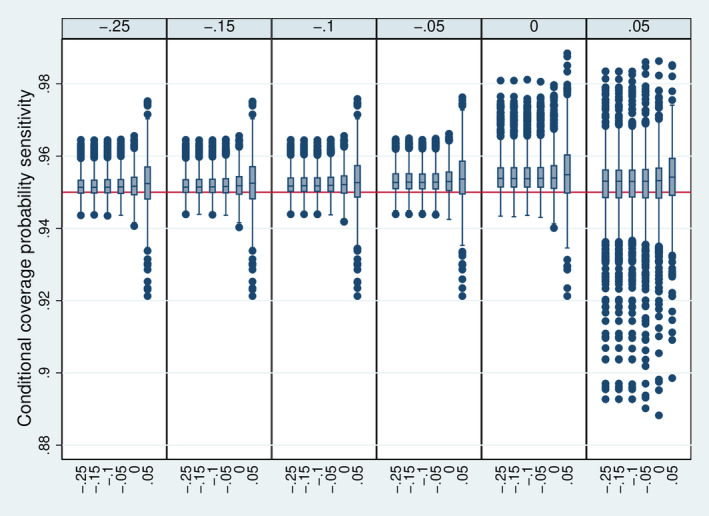
Distribution of the conditional coverage probability for sensitivity overall 31104 simulation designs stratified by $\Delta_{\mathrm{se}}$ (top numbers) and $\Delta_{\mathrm{sp}}$ (bottom numbers). However, 1,594 designs with a stopping frequency above 0.975, that is, less than 250 final analyses performed, are omitted.

**FIGURE B5:**
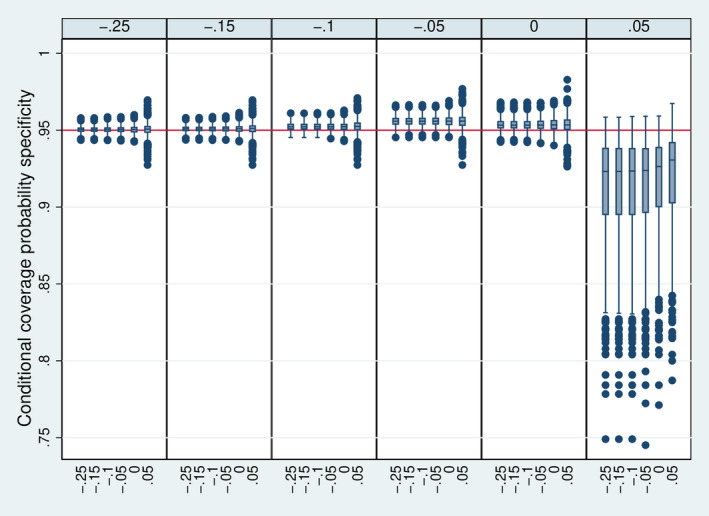
Distribution of the conditional coverage probability for specificity overall 31104 simulation designs stratified by $\Delta_{\mathrm{sp}}$ (top numbers) and $\Delta_{\mathrm{se}}$ (bottom numbers). However, 1,594 designs with a stopping frequency above 0.975, that is, less than 250 final analyses performed, are omitted.

**FIGURE B6:**
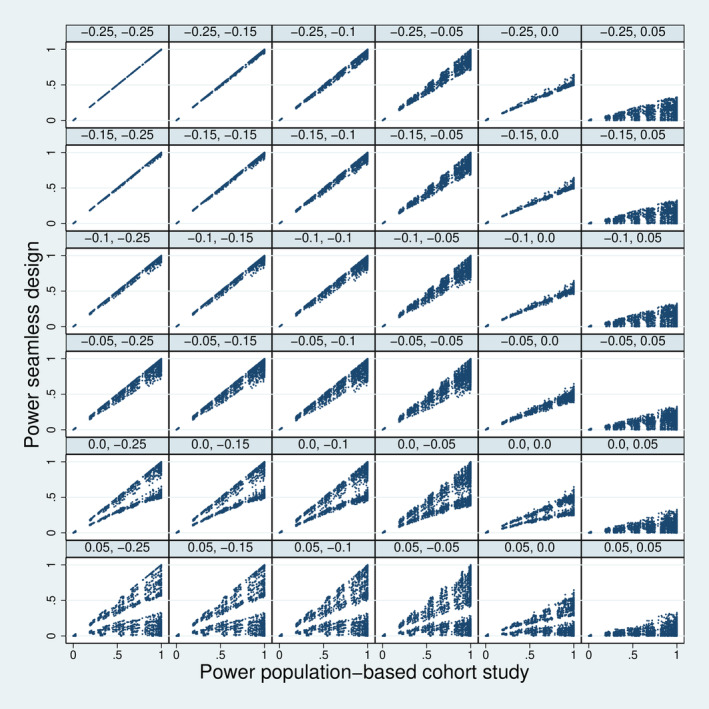
The joint distribution of the power of the seamless design and the power of a population‐based cohort study overall 31104 simulation designs combined with all 25 choices for $se_{0}$ and $sp_{0}$ stratified by $\Delta_{\mathrm{sp}}$ (columns) and $\Delta_{\mathrm{se}}$ (rows).

## Data Availability

The data that support the findings of this study are available from the corresponding author upon reasonable request.

## Appendix A Two Approaches to Determine the Stopping Time s

Usually, the sample size in early case–control studies is chosen for pragmatic reasons, not least because no prior knowledge of the parameters of sample size planning exists. To determine the stopping time s, we consider here two alternative approaches. For both approaches, we use the following nomenclature: Let N denote the intended sample size for the final analysis and let s denote the stopping time as a fraction of this sample size. Furthermore, the prevalence is denoted by p, leading to the final number of individuals with a target condition for sensitivity and without a target condition for specificity:

$$n_{\mathrm{se}}=N\cdot p\;\text{and}\;\;n_{\mathrm{sp}}=N\cdot \left(1-p\right).$$

If p≤0.5, $n_{\mathrm{se}}$ is the smaller subgroup, otherwise it is $n_{\mathrm{sp}}$.

1. One possibility is to specify the proportion of the smaller subgroup to be already collected in stage I ($n_{\mathrm{se},1}$ or $n_{\mathrm{sp},1}$, without additional cases or controls), denoted by $q=n_{\mathrm{se},1}/n_{\mathrm{se}}$(if p≤0.5) or $q=n_{\mathrm{sp},1}/n_{\mathrm{sp}}$ (if p>0.5), then s can be calculated as follows:

s=q·2·(1−p)ifp≤0.5and

s=q·2·pifp>0.5,respectively.

Some example calculations are provided in Table A1, which also shows that the choice of q may be limited by the number of additional cases (or controls) available.

2. Another possibility is to require the same number of cases (if p<0.5, controls otherwise) in the interim analysis as in the final analysis, as both analyses address the same question. The expected number of cases in the final analysis is p·N, and when stopping at time s, the expected number of already existing controls is (1−p)·s·N, and the number of cases will be enriched in order to match this number. Consequently, the intended equality is reached if p·N=(1−p)·s·N, implying s=p/(1−p). For p equal to 0.3,0.25,0.2,0.15,0.1 the corresponding values of s are 0.43,0.33,0.25,0.18, and 0.11. Additional considerations may refer to the absolute number of cases to be added. This number is equal to (1−2·p)·s·N. If the number of available additional cases is limited, this may decrease the choice of s.

**TABLE A1:** Some example constellations of the different parameters determining the sample size of stage 1 and the needed number of additional cases.

			For N=1000				
Prevalence p	Proportion q	Stopping time s	$n_{\mathrm{se}}$	$n_{\mathrm{sp}}$	$n_{\mathrm{se},1}$	$n_{\mathrm{sp},1}$	Additional cases
0.1	0.1	0.18	100	900	10	90	80
0.1	0.2	0.36	100	900	20	180	160
0.1	0.3	0.54	100	900	30	270	240
0.2	0.1	0.16	200	800	20	80	60
0.2	0.2	0.32	200	800	40	160	120
0.2	0.3	0.48	200	800	60	240	180
0.3	0.1	0.14	300	700	30	70	40
0.3	0.2	0.28	300	700	60	140	80
0.3	0.3	0.42	300	700	90	210	120

## Appendix B Results From an Additional Simulation Study

In this simulation study, we considered the values −0.25,−0.15,−0.1,−0.05,0.0, and 0.05 for the difference between the true values and the futility boundaries, that is, for $\Delta_{\mathrm{se}}$ and $\Delta_{\mathrm{sp}}$. All possible 36 combinations were considered. Otherwise, the set‐up of the simulation study was identical to the one described in Table 2. However, only 10000 instead of 40000 repetitions were performed.

Figure B1 illustrates that the relative bias of the sensitivity estimate is rather independent of $\Delta_{\mathrm{sp}},$ and Figure B2 illustrates that the relative bias of the specificity estimate is rather independent of $\Delta_{\mathrm{se}}$. Consequently, all conclusions of the main part of the paper derived for the special case $\Delta_{\mathrm{se}}=\Delta_{\mathrm{sp}},$ apply also here. Similarly, the joint distribution of relative bias and stopping frequency depicted in Figure B3 is similar to the one shown in Figure 5, supporting the same conclusions.

Figure B4 illustrates that the conditional coverage probability for sensitivity is rather independent of $\Delta_{\mathrm{sp}},$ and Figure B5 illustrates that the conditional coverage probability of specificity is rather independent of $\Delta_{\mathrm{se}}$. Consequently, all conclusions of the main part of the paper derived from the special case $\Delta_{\mathrm{se}}=\Delta_{\mathrm{sp}}$ apply also here. From Figure B6, we can conclude that the values of both $\Delta_{\mathrm{se}}\;\text{and}\;\Delta_{\mathrm{sp}}$ of −0.1 or below imply nearly a protection against a loss of power, extending the results obtained in the main part of the paper.
